# Comparative Analysis of Heller Myotomy With Dor Versus Toupet Fundoplication for Achalasia Cardia

**DOI:** 10.7759/cureus.30243

**Published:** 2022-10-13

**Authors:** Sunita Suman, Vaibhav K Varshney, Subhash Soni, Sanjeev Sachdeva, Sabir Hussain, Narendra Bhargava

**Affiliations:** 1 Surgical Gastroenterology, All India Institute of Medical Sciences, Jodhpur, Jodhpur, IND; 2 Department of Gastroenterology, Govind Ballabh Pant Postgraduate Institute of Medical Education and Research, New Delhi, IND; 3 Department of Gastroenterology, Dr. Sampurnanand Medical College, Jodhpur, IND

**Keywords:** high-resolution esophageal manometry, heath-related quality of life, ph study, dor fundoplication, heller myotomy, achalasia cardia

## Abstract

Background

Heller myotomy (HM) with partial fundoplication is the standard of care for achalasia cardia. However, the choice of partial fundoplication is controversial. In this study, we compared both types of fundoplication concerning subjective and objective parameters.

Methodology

This prospective comparative study comprised a total of 30 consecutive patients who underwent laparoscopic/robotic HM with either Dor fundoplication (DF) (n = 15) or Toupet fundoplication (TF) (n = 15). Preoperative baseline characteristics, intraoperative details, and postoperative complications were recorded. Patients were followed with Eckardt score, quality of life-related scores, 24-hour pH study, and high-resolution manometry (HRM) at the one-year follow-up.

Results

There was no significant difference between the two groups regarding preoperative baseline parameters, length of hospital stay, and postoperative complications. The HM+DF group had four (27%) patients with recurrence/failure with none in the HM+TF, but it was not significant (p = 0.79). Symptom scores were similar between the groups at six and 12 months of follow-up. One patient in the HM+DF group and two in the HM+TF group had significant pathological acid reflux (p = 0.483). On HRM, HM+TF showed a trend toward significance in terms of esophagogastric junction (EGJ) relaxation (p = 0.058) with a non-significant difference in median integrated relaxation pressure (p = 0.081).

Conclusions

The study showed a trend toward lower failure rates and improved EGJ relaxation with similar reflux rates in patients who underwent HM+TF compared to HM+DF. However, long-term follow-up is required to validate our findings with well-defined subjective and objective criteria.

## Introduction

Achalasia is a disease involving the esophagus which causes progressive degeneration of nerves leading to aperistalsis of the esophagus and failure of relaxation of the lower esophageal sphincter (LES). Because the disease process involves a gradual loss of ganglion cells in Auerbach’s plexus of the esophagus, it is essentially irreversible [1]. Consequently, the management options, without exception, are palliative in nature. With the widespread adoption of minimally invasive techniques, both laparoscopic Heller myotomy (LHM) and robotic Heller myotomy (RHM) were expeditiously embraced worldwide. However, with the increasing popularity of Heller myotomy (HM), evidence is accumulating regarding the risk of postoperative reflux [2,3].

Partial fundoplication is preferred after HM as it is reported that there is an increased risk of dysphagia, with no significant difference in pathological reflux after a total fundoplication [4]. Further, Rawlings et al., in their randomized study, demonstrated the equivalence of Dor fundoplication (DF) and Toupet fundoplication (TF) after LHM regarding symptom control and postoperative reflux [5]. However, a meta-analysis by Wei et al. of two randomized control trials (RCTs) and four non-RCTs found a statistically higher risk of reflux in the DF group compared with the other fundoplication group [6].

Our study aimed to compare postoperative reflux and dysphagia in patients undergoing HM and partial fundoplication with a 24-hour pH study, high-resolution manometry (HRM), and the quality of life outcome questionnaires. Integration of objective and subjective assessment of postoperative reflux may aid surgeons in selecting the ideal anti-reflux procedure and prevent complications related to asymptomatic reflux after esophageal myotomy.

## Materials and methods

This prospective study was conducted in the Department of Surgical Gastroenterology at the All India Institute of Medical Sciences (AIIMS), Jodhpur (India), to compare the incidence of reflux between DF and TF in achalasia cardia after HM, as well as to determine the improvement in the quality of life. All patients undergoing minimally invasive HM with partial fundoplication from July 2018 to December 2019 were included in the study and were followed up till December 2020. Ethical clearance was attained from the Institutional Ethics Committee with reference number AIIMS/IEC/2018/777.

The sample size was calculated by assuming a surgical complication proportion from the study by Torres-Villalobos et al. [7]. The pathologic reflux of 0.10 and 0.04 in two groups with a risk difference of 0.06 and absolute precision of 80% along with a clinically significant level of 0.05 was considered [7]. A sample size of 12 patients per group was calculated using the following formula: n = Z21-a/2[P1(1 - P1) + P2(1 - P2)]/d2 (P1: proportion in the first group; P2: proportion in the second group; d2: population risk difference; 1-a: desired confidence level). With a dropout rate of about 10%, 15 patients were required to be included in each group.

Patients with end-stage achalasia cardia/sigmoid esophagus, previous upper abdominal surgery or peroral endoscopic myotomy (POEM), prior HM, Chagas disease or other esophageal motility disorders, uncorrectable coagulopathy, and significant comorbid conditions precluding the use of general anesthesia/American Society of Anesthesiologist (ASA) class 3-4 were excluded from the study.

Clinical history of dysphagia, chest pain, chronic cough, nocturnal cough, dyspnea on exertion, and weight loss was obtained. Eckardt score and achalasia-specific quality of life (ASQOL) and gastroesophageal reflux disease health-related quality of life (GERD-HRQL) questionnaires were filled in the preoperative setting. Chicago classification of esophageal motility disorders, version 3.0, was used for defining achalasia cardia, and the diagnosis was confirmed with an oral contrast study (OCS), HRM, and upper gastrointestinal endoscopy (UGIE).

Laparoscopic and robotic HM was performed with either DF or TF. The myotomy was initiated just above the esophagogastric junction (EGJ) and extended 4-5 cm proximally, followed by 2-3 cm distally onto the gastric side, such that a total myotomy length of 6-8 cm was achieved. The choice between anterior and posterior fundoplication was the surgeon’s preference. Postoperatively, patients were started on oral liquids on day one or two after OCS, and the nasogastric tube and abdominal drain were usually removed on postoperative day (POD) two or three. Patients were typically discharged by POD three or four. Patients were followed up every six months for one year, either via a physical visit to the outpatient department or telephonically, and re-assessed by the physician with the Eckardt, ASQOL, and GERD-HRQL questionnaires. At the one-year follow-up, a 24-hour pH study and manometry were done to assess reflux and dysphagia objectively.

Failure was defined as a persistent Eckardt score of >3, and recurrence was described as a decrease with an Eckardt score of <3 with an increase to >3 on follow-up. Pathological reflux was defined as a DeMeester score of more than 14.7 on a 24-hour pH study.

Statistical analysis

Data were acquired in a specified format as in a proforma and entered in SPSS version 27 (IBM Corp., Armonk, NY, USA)/Microsoft Excel software for analysis. Measured data were expressed as median with interquartile range (IQR) at the 25th and 75th percentiles or as percentages. Chi-square or Fisher’s exact test was used for comparing proportions, whichever was applicable. Numerical data were compared using the Mann-Whitney U test. Pre- and post-intervention scores were compared using the Wilcoxon signed-rank test. A two-tailed p-value of 0.05 or less was considered statistically significant.

## Results

Both HM+DF (n = 15) and HM+TF (n = 15) groups had similar baseline characteristics (Table 1). The primary presentation was dysphagia to liquids and solids in both groups, and the majority (93.3%) also had accompanying regurgitation. Weight loss was noticed in 60% and 73% of HM+DF and HM+TF groups, respectively. Preoperative symptoms associated with achalasia, as assessed with Eckardt and ASQOL scores, were similar between the groups. Most patients had type II achalasia (Chicago classification) except for three patients, one in each group who was classified as type I achalasia and one in the HM+TF group who had type III achalasia. Median integrated relaxation pressure (IRP) (IQR) was 23.8 (20.1, 26.45) for the HM+DF group and 27 (21.95, 30.45) for the HM+TF group (p = 0.144). Preoperatively, pneumatic dilatation was done in four patients in the HM+DF group and two in the HM+TF group. However, symptoms recurred within six months, and the patients were referred for further intervention.

**Table 1:** Baseline characteristics. IQR: interquartile range; BMI: body mass index; GERD-HRQL: gastroesophageal reflux disease health-related quality of life

Characteristics	Dor (n = 15)	Toupet (n = 15)	P-value
Age, years [median (IQR)]	27 (24, 44)	36 (29, 45)	0.205
Gender			
Male, n (%)	10 (66.7)	8 (53.3)	0.710
Female, n (%)	5 (33.3)	7 (46.7)	
Presenting symptoms			
Duration, months [median (IQR)]	36 (24,60)	36 (29,45)	0.867
Dysphagia, n (%)	15 (100)	15 (100)	-
Regurgitation, n (%)	14 (93.3)	14 (93.3)	1.000
Chest pain, n (%)	2 (13.3)	1 (6.7)	1.000
Heartburn, n (%)	2 (13.3)	1 (6.7)	1.000
Weight loss, n (%)	9 (60)	11 (73.3)	0.700
Respiratory complaints, n (%)	6 (40)	2 (13.3)	0.215
Previous treatment n (%)			
Medical	3 (20)	3 (20)	1.000
Endoscopic	4 (26.7)	2 (13.3)	0.651
Comorbidities, n (%)	1 (6.67)	1 (6.67)	1.000
BMI, kg/m^2^ [median (IQR)]	19.08 (16.5, 20.2)	20.2 (18.04, 21.3)	0.125
Hemoglobin, g/dL [median IQR)]	12.8 (11.4, 15)	13.6 (11.7, 14.5)	0.787
Serum albumin, g/dL [median (IQR)]	4.3 (4, 4.58)	4.38 (4, 4.67)	0.480
Eckardt score [median (IQR)]	8 (7, 9)	7 (6, 8)	0.214
Achalasia quality of life [median (IQR)]	54 (50, 62)	52 (50, 57)	0.917
GERD-HRQL [median (IQR)]	11 (0, 14)	9 (2, 17)	0.738
Heartburn score [median (IQR)]	0 (0, 0)	0 (0, 0)	0.150
Regurgitation score [median (IQR)]	9 (0, 12)	6 (0, 12)	0.329

All patients underwent minimally invasive surgery, and there was no conversion to open surgery. Eleven (73.3%) and four (26.7%) patients in each group underwent laparoscopic and robotic HM, respectively. The length of myotomy on the esophagus and stomach and the median hospital stay was similar between the two groups. None of our patients required readmission. Although three patients in the HM+DF group had a mucosal tear, none had clinical leaks, and all recovered uneventfully (Table 2).

**Table 2:** Intraoperative parameters and postoperative outcomes. IQR: interquartile range; POD: postoperative day

Characteristics	Dor (n = 15)	Toupet (n = 15)	P-value
Type of surgery			
Laparoscopic, n (%)	11 (73.3)	11 (73.3)	0.651
Robotic, n (%)	4 (26.7)	4 (26.7)	
Mucosal perforation, n (%)	3 (20)	0 (0)	0.224
Length of myotomy			
Esophagus, cm [median (IQR)]	5 (5, 5)	5 (4, 5)	0.203
Stomach, cm [median (IQR)]	2 (2, 3)	2 (2, 2)	0.115
Duration of surgery, hours [median (IQR)]	120 (120, 150)	120 (90, 150)	0.155
Postoperative length of stay, days [median (IQR)]	3 (2, 5)	3 (2, 3)	0.197
Nasogastric tube removal, POD [median (IQR)]	2 (1, 2)	1 (1, 2)	0.155
Abdominal drain removal, POD [median (IQR)]	3 (2, 4)	2 (2, 3)	0.065
Oral contrast study, POD [median (IQR)]	2 (1, 2)	1 (1, 2)	0.082
Initiation of oral feed, POD [median (IQR)]	2 (1, 3)	1 (1, 2)	0.065
Dysphagia, n (%)	1 (6.7)	0 (0)	1.000

The overall success rate of minimally invasive myotomy was 86.7%. Although there was more failure/recurrence (4, 26.7%) in the HM+DF group compared to the HM+TF group (0%), this difference was not significant (p = 0.799). The Eckardt, ASQOL, and GERD-HRQL scores were not significantly different between the groups (p = 0.751, 0.915, 0.520, respectively) at the one-year follow-up (Table 3).

**Table 3:** Comparison of subjective assessment of dysphagia and reflux at six and 12 months after surgery. IQR: interquartile range; GERD-HRQL: gastroesophageal reflux disease health-related quality of life

Outcomes	Dor (n = 15) (six months)	Toupet (n = 15) (six months)	P-value (six months)	Dor (n = 15) (one year)	Toupet (n = 15) (one year)	P-value (one year)
Eckardt score, median [(IQR)]	0 (0,4)	0 (0,1)	0.799	0 (0,4)	1 (0,1)	0.751
Remission, n (%)	11 (73.3)	15 (100)		11 (73.3)	15 (100)	
Failure, n (%)	4 (26.7)	0 (0)		4 (26.7)	0 (0)	
Achalasia quality of life [median (IQR)]	14 (14, 33)	14 (0, 23)	0.347	14 (0, 33)	14 (0, 23)	0.915
GERD-HRQL [median (IQR)]	0 (0, 1)	0 (0, 0)	0.520	0 (0, 1)	0 (0, 0)	0.520
Heartburn score [median (IQR)]	0 (0, 0)	0 (0, 0)	1.000	0 (0, 0)	0 (0, 0)	0.317
Regurgitation score [median (IQR)]	0 (0, 1)	0 (0, 0)	0.142	0 (0, 1)	0 (0, 0)	0.142

The difference between the two groups in the 24-hour pH study was also insignificant. Two patients in the TF group and only one in the DF group had a DeMeester score of more than 14.7 (p = 0.483). On manometry, median IRP (IQR) was 16.4 (11, 22.9) mmHg in the HM+DF group and 10 (3.1, 12.35) mmHg in the HM+TF group (p = 0.081). EGJ relaxation was incomplete in eight patients in the HM+DF group and three in the HM+TF group. This difference seemed to tend toward significance (p = 0.058) (Table 4).

**Table 4:** Objective assessment of dysphagia and reflux at 12 months after surgery. IQR: interquartile range; EGJ: esophagogastric junction; IRP: integrated relaxation pressure

At one-year follow-up	Dor (n = 15)	Toupet (n = 15)	P-value
24-hour pH study			
% upright time in reflux [median (IQR)]	0 (0, 11.93)	0.14 (0, 3.36)	0.546
% recumbent time in reflux [median (IQR)]	0 (0, 3.15)	0.06 (0, 3.7)	0.436
% total time in reflux, minutes [median (IQR)]	0 (0, 5.78)	1.4 (0, 2.85)	0.409
Episodes >5 minutes [median (IQR)]	0 (0, 1.35)	1 (0, 2)	0.288
Longest episode in minutes [median (IQR)]	0 (0, 87.45)	1.3 (0, 11.27)	0.620
Total episodes [median (IQR)]	0 (0, 1.35)	1.3 (0, 11.27)	0.229
DeMeester score [median (IQR)]	0.8 (0.8, 25.48)	1.1 (0, 14.6)	0.680
DeMeester score >14.7, n (%)	1 (6.7)	2 (13.3)	0.483
Manometry			
Median IRP [median (IQR)]	16.4 (11, 22.9)	10 (3.1, 12.35)	0.081
Basal EGJ pressure			
Normal, n (%)	7 (46.7)	4 (26.7)	0.255
Low, n (%)	8 (53.3)	11 (73.3)	
EGJ relaxation			
Present, n (%)	7 (46.7)	12 (80)	0.058
Incomplete, n (%)	8 (53.3)	3 (20)	
Mean expiratory EGJ pressure [median (IQR)]	9.95 (0.97, 18.52)	7.6 (5.45, 11.7)	1.000
Mean inspiratory EGJ pressure [median (IQR)]	13.3 (7.9, 23.7)	13.4 (10.4, 21.7)	0.782

We did not find any significant difference between the Eckardt, ASQOL, and GERD-HRQL scores between six and 12 months of follow-up, implying that remission was maintained at the one-year follow-up (Table 5).

**Table 5:** Comparison of outcomes between preoperative, six months, and 12 months. HM+DF: Heller myotomy with Dor fundoplication; HM+TF: Heller myotomy with Toupet fundoplication; IQR: interquartile range; GERD-HRQL: gastroesophageal reflux disease health-related quality of life

Factors	Preoperative score [median (IQR)]	Score at six months [median (IQR)]	Score at 12 months [median (IQR)]	P-value (preoperative and six months)	P-value (preoperative and 12 months)	P-value (six and 12 months)
HM+DF						
Eckardt score	8 (7, 9)	0 (0, 4)	0 (0, 4)	0.001	0.001	0.317
Achalasia quality of life	54 (50, 62)	14 (14, 33)	14 (0, 33)	0.001	0.001	0.083
GERD-HRQL	11 (0, 14)	0 (0, 1)	0 (0, 1)	0.016	0.016	1.000
Heartburn score	0 (0, 0)	0 (0, 0)	0 (0, 0)	0.317	0.317	1.000
Regurgitation score	9 (0, 12)	0 (0, 1)	0 (0, 1)	0.006	0.006	1.000
HM+TF						
Eckardt score	7 (6, 8)	0 (0, 1)	0 (0, 1)	0.001	0.001	0.664
Achalasia quality of life	52 (50, 57)	14 (0, 23)	14 (0, 23)	0.001	0.001	0.655
GERD-HRQL	9 (2, 17)	0 (0, 0)	0 (0, 0)	0.002	0.002	0.180
Heartburn score	0 (0, 0)	0 (0, 0)	0 (0, 0)	0.180	0.180	1.000
Regurgitation score	6 (0, 12)	0 (0, 0)	0 (0, 0)	0.003	0.003	0.317

On multivariate analysis, none of the preoperative factors was predictive of failure or recurrence (Table 6).

**Table 6:** Factors associated with failure/recurrence. BMI: body mass index; EGJ: esophagogastric junction; IRP: integrated relaxation pressure

	Remission (26)	Failure (4)	P-value
	Median (Q1-Q3)	Median (Q1-Q3)	
Age	32 (27-44)	25.5 (24-35.5)	0.271
Symptom duration	42.0 (24-60)	30.0 (14.5-60)	0.536
BMI	20.0 (17-21.2)	18.9 (17.6-19.6)	0.391
Mean expiratory EGJ pressure	43.0 (41.1-45.8)	41.5 (32.1-43)	0.157
Mean inspiratory EGJ pressure	44.9 (43.1-48.4)	43.5 (36.5-45.1)	0.328
Median IRP	24.7 (23.1-27)	25 (24.4-25.2)	1.000
Duration	120 (100-150)	120 (120-135.5)	0.659
Myotomy esophagus	5 (5-5)	5 (4.5-5)	0.976
Myotomy stomach	2 (2-2)	2 (1.5-3)	0.617
Eckardt score	8 (6-8)	8 (7-9)	0.425
Achalasia quality of life	22 (20-24)	22.5 (19.5-25.5)	0.883
GERD-HRQL	9.5 (8-15)	11.5 (10-13)	0.791
Regurgitation score (10–15)	6 (6-12)	10.0 (8.5-11.5)	0.391

## Discussion

Achalasia cardia is an esophageal motility disorder of undetermined etiology characterized by progressive and selective neurodegeneration of myenteric inhibitory neurons. These patients have esophageal aperistalsis with failure of relaxation of LES after swallowing. The primary aim of treatment is palliative with the alleviation of functional obstruction. As the benefits associated with HM and adjunct partial fundoplication rely on the delicate balance between myotomy for dysphagia and fundoplication to overcome the resulting acid reflux, several parameters need to be assessed. However, the choice of partial fundoplication remains contentious. We evaluated the difference between these procedures based on patient-reported outcomes (subjective) and objective assessments of both dysphagia and reflux.

All patients in our study underwent minimally invasive surgery, either laparoscopic or robotic-assisted. Several retrospective studies have shown equivalent outcomes in comparing LHM and RHM with respect to operative time, complications, and success rates [8]. The length of myotomy in our study was 4-5 cm toward the esophageal side and 2-3 cm toward the stomach. Few studies have shown that the length of myotomy on the stomach >2.5 cm results in a greater degree of alleviation of dysphagia symptoms, albeit with a higher rate of reflux [9]. Others have shown benefits with an extended myotomy of 3 cm [10]. However, it is reported that the distensibility index (DI) increased till a myotomy of 2 cm distal to EGJ, beyond which DI did not improve [11]. Further, the mucosal perforation rate was seen in 10% of patients, similar to that reported in the literature [12,13]. We performed DF intentionally in such cases with no postoperative complications.

Our patients were followed up at six months and then again at 12 months after surgery, with subjective and objective assessments of dysphagia and reflux. The International Society for Diseases of the Esophagus (ISDE) guidelines recommend evaluating symptomatic improvement as the best measure of success or failure of a treatment modality [14]. The Eckardt score is the most commonly used measure for the evaluation of treatment success, although other scores have also been used. The majority of our patients had Eckardt scores <3. Four patients in the HM+DF group had an Eckardt score of 4 and were considered treatment failure/recurrence, whereas none of the patients in the HM+TF group had treatment failure. This difference did not reach statistical significance in our study and corroborated with the published literature [15]. Studies have shown that using the Eckardt score as the sole predictor of the surgical outcome can overestimate success rates [16].

Further, few studies have shown that TF is associated with lower recurrence rates than DF [17]. This difference may be explained by the traction caused by the posterior fundoplication that prevents restenosis/stricture. The causes of failure could be explained by inadequate myotomy, scarring, a stricture induced by acid reflux, or a tight fundoplication. All patients who had achieved remission at six months continued to be in remission at the 12-month follow-up. There was no significant difference in the Eckardt score between six and 12 months of follow-up. Out of the four patients, two were advised pneumatic dilatation; however, the other two refused any further intervention in view of the ongoing coronavirus disease 2019 pandemic and were kept on regular telephonic follow-up.

Health-related quality of life is an essential tool in the assessment of functional disorders, but only a few studies in the literature report quality of life in achalasia patients [5,7,18]. There is a lack of a universally acceptable questionnaire for evaluation. Various studies have used heterogeneous questionnaires, many of which are not specific to the disease and are used in a generalized way. We used ASQOL, a well-validated score reported by Urbach et al. [19], and this questionnaire can fill the void in the available literature. ASQOL scores improved significantly from the preoperative to the postoperative period at six and 12 months of follow-up, with a median score of 14 (range: 0 to 33) for the HM+DF group and 14 (range: 0 to 23) for the HM+TF group at one year, with no difference between these groups. The scores were similar to those reported in the literature [20]. Slone et al. compared the Eckardt score and ASQOL and reported that an ASQOL score cut-off of 15, with a correlation coefficient of 0.85 with the Eckardt score, was 87% accurate in identifying success [21].

GERD-HRQL is an accepted and validated questionnaire for the assessment of GERD symptoms [22]. We employed this questionnaire for the subjective evaluation of gastroesophageal reflux symptoms of patients after HM and partial fundoplication. However, none of our patients presented with symptoms of reflux. All our patients underwent a 24-hour pH study to evaluate pathological reflux objectively. Pathological reflux was seen in one patient in the HM+DF group and two in the HM+TF, which was not a significant difference. This is in accordance with the available literature [7]. As our study shows, pathological acid reflux is seldom symptomatic, exposing undiagnosed patients to a higher risk of continuous acid exposure, leading to Barrett’s esophagus or even malignancy. The true incidence of GERD is often underestimated by subjective assessment in patients who undergo HM [16,23,24]. All patients after an HM should be followed up with an ambulatory 24-hour pH study at the one-year follow-up.

To objectively evaluate relief in dysphagia, we also performed HRM in our patients one year after surgery. The postoperative median IRP was higher in the HM+DF group than in the HM+TF group, but this difference was not statistically significant. This finding is akin to the results reported by Torres-Villalobos et al. [7]. They found a median IRP of 14.8+8.62 for the HM+DF group and 7.21+5.84 for the HM+TF group at six months of follow-up, although greater in the DF group, it was not statistically significant. We found a trend toward significance in favor of HM+TF in terms of EGJ relaxation. However, the clinical importance and relevance of the difference in this parameter need to be evaluated on further follow-up. To our knowledge, we could not find any other studies that reported EGJ relaxation during postoperative evaluation.

Nevertheless, our study is fraught with limitations of the small sample size, selection bias, and a short postoperative follow-up. Additionally, postoperative endoscopy was not performed routinely unless symptomatic. However, our study is unique as it evaluated ASQOL, improvement in manometric outcomes concerning dysphagia, and assessment of reflux with the GERD-HRQL questionnaire, as well as an ambulatory 24-hour pH study together in follow-up. We believe that only symptomatic assessment may lead to underdiagnosis of postoperative GERD and its complications; hence, objective evaluation with a 24-hour pH study is essential.

## Conclusions

HM with partial fundoplication is currently the standard of care for patients with achalasia. Although the choice of fundoplication may be decided based on an individual surgeon’s preference, our study shows a trend toward lower failure rates and improved EGJ relaxation with similar pathological reflux rates in patients who underwent HM+TF compared to those who underwent HM+DF. Poor clearance of asymptomatic acid refluxate in a dilated aperistaltic esophagus of this chronic incurable disease is a matter of grave concern in the long term. We believe that future clinical research for ideal fundoplication post-myotomy should focus on objective reflux parameters rather than relying solely on symptoms during follow-up. Larger studies with longer follow-ups aimed at comparing the two fundoplications with a 24-hour pH study at follow-up may end the surgeon’s dilemma.
